# Post-tonsillectomy hemorrhage: Incidence and predictors from a two-year retrospective study

**DOI:** 10.6026/973206300221314

**Published:** 2026-03-31

**Authors:** Gunjan Virendra Manchanda, Debasis Jena, Amiya Kumar Nayak, Rahul Tiwari, Maitri Vipulkumar Doshi, Akriti Mahajan, Heena Dixit, Afroz Kalmee Syed

**Affiliations:** 1 Department of Otorhinolaryngology, Government Medical College and Maharashtra Post Graduate Institute of Medical Education and Research (MUHS), Nashik, Maharashtra, India; 2 Department of ENT, SCB Medical College, Cuttack, Odisha, India; 3 Department of Anesthesiology, PRM Medical college, Baripada, Odisha, India; 4 Department of Oral and Maxillofacial Surgery, Narsinhbhai Patel Dental College and Hospital, Sankalchand Patel University, Visnagar, Gujarat, India; 5 University Geomedi LLC, Tbilisi, Georgia; 6 Department of Oral medicine and Radiology, Desh Bhagat Dental College and Hospital, Mandi Gobindgarh, Punjab, India; 7 Department of Medical Health Administration, Index Institute, Malwanchal University, Index City, Nemawar Road, Indore, Madhya Pradesh, India; 8 Department of Oral and Maxillofacial Pathology, Scientific Medical Writer (Writing and Publications), Tenali andhra Pradesh, India

**Keywords:** Tonsillectomy, postoperative hemorrhage risk factors, retrospective study, otolaryngology

## Abstract

Post-tonsillectomy hemorrhage (PTH) remains a clinically significant and potentially life-threatening complication and variability in reported incidence and predictors across institutions continues to challenge standardized risk stratification and perioperative management. The two-year study retrospectively assessed the frequency and severity of postoperative bleeding at the tertiary otolaryngology centre. Operative and clinical variables were examined to determine the factors related to secondary and primary hemorrhage. The hemorrhage rate overall was in line with the current population-based research. Multivariable analysis showed age, surgical indications and the use of perioperative medications as significant predictors.

## Background:

Tonsillectomy is one of the most commonly performed otolaryngology procedures worldwide, yet postoperative hemorrhage continues to represent a potentially life-threatening complication. Recent population-based studies have reported post-tonsillectomy hemorrhage (PTH) rates ranging from 2% to 7%, with higher risk in adolescents and adults [1]. Age-dependent variation in bleeding risk has been demonstrated in national database analyses [1]. Surgical indication also influences hemorrhage incidence, with recurrent tonsillitis carrying greater risk compared with obstructive sleep-disordered breathing [2]. Pharmacologic factors such as perioperative non-steroidal anti-inflammatory drug use have been associated with increased postoperative bleeding in meta-analytic evidence [3]. Emerging trials have explored antifibrinolytic strategies such as tranexamic acid to mitigate bleeding risk [4]. Identification of institution-specific incidence and predictors is essential to optimize perioperative protocols and counseling. Therefore, it is of interest to report the incidence, timing and clinical predictors of post-tonsillectomy hemorrhage in a tertiary referral center over a two-year period.

## Materials and Methods:

An observational retrospective study carried out in the Department of Otolaryngology of a teaching hospital of tertiary treatment. The ethics committee of the institution's approval had to be obtained before collecting data. Medical documents of all patients undergoing elective tonsillectomy with or without adenoidectomy between January 2023 to December 2024 were examined. Patients suffering from coagulopathies that were known or syndromic craniofacial abnormalities or records that were not complete were ruled out. The data extracted comprised gender, age or surgical indication, the operating procedure (cold cut or bipolar) and perioperative analgesic protocols and the occurrence in postoperative hemorrhage. PTH was classified as primary (within 24 hours) and secondarily (after 24-hours) as per the definitions in current research [5]. A hemorrhage that required and the emergency department to visit or readmission, as well as surgical hemostasis were deemed to be clinically important. Descriptive statistics were developed to determine basic characteristics. The incidence of PTH was calculated as a proportion of tonsillectomies in total. Potential predictors of hemorrhage were evaluated with the chi-square test along with multiple logistic regressions. Statistical significance was defined as p less than 0.05. Analyses were carried out with SPSS Version 26.0.

## Results:

The results of 412 procedures have been analyzed. The majority of the patients were young adults and adolescents, with median years that were 14.8 years. Tonsillitis recurrent was the most common reason for surgical intervention. Dissection with cold was still the preferred surgical procedure. The distribution of the operative and demographic variables was similar to the most recent study of cohorts across the globe that examined the current practices for tonsillectomy Table 1 (see PDF). Post-tonsillectomy hemorrhage occurred in 5.3% of cases, consistent with modern epidemiological data. Secondary hemorrhage accounted for the majority of bleeding episodes. Adolescents demonstrated significantly higher bleeding risk compared with younger children. Surgery for recurrent tonsillitis and perioperative NSAID administration were independent predictors of hemorrhage. Operative technique did not significantly influence bleeding risk. These findings align with recent multi-center retrospective studies and meta-analyses evaluating PTH risk factors Table 2 (see PDF).

## Discussion: 

A clinically significant PTH rate of 5.3% was found in this series which is similar to modern international studies reporting a rate between 3% and 7% [6, 7]. One large North American population cohort showed that age was a powerful predictor of hemorrhaging and peripubertal children had much higher PTH incidence than young children [1]. This age-related susceptibility was reinforced in the present study population, which provides support for the potential influence of physiological and behavioral conditions on postoperative wound integration. Surgical indication was a significant predictor and recurrent tonsillitis with increased risk of more bleeding compared to sleep-disordered breathing type. This finding is in line with that of recent retrospective cohort evidence, which indicated the enhanced inflammation-related vascularity in patients with chronic tonsillitis [8]. The higher secondary hemorrhage rate in our series also contrasts to published evidence that sloughing of tonsillar eschar as the main cause beyond Po day 1 [9]. Use of NSAIDs in the perioperative period was a statistically significant factor for bleeding. A recent meta-analysis looking at non-opioid analgesic treatment post tonsillectomy, demonstrated a significant increased odds ratio of developing a postoperative hemorrhage with the use of NSAID [3]. On the other hand, recent RCTs of tranexamic acid have reported that postoperative bleeding has decreased without increase in the incidence of adverse events [4, 10]. These results indicate that antifibrinolytic prophylaxis may be a useful adjunct in high-risk patients the type of operative technique used had no impact on bleeding in our analysis. Contemporary comparative studies of cold dissection and electrocautery have also routinely found the incidence of PTH is similar when surgeon experience as well as hemostasis protocols are controlled [11]. As such, patient factors - not technical variables likely play a greater role in bleeding risks. The retrospective nature of the study was considered a limitation. Nevertheless, in the absence of such clinical evidence from institutional practice is still necessary to direct quality improvement work. Recent high-level national database analyses highlight that local audit is so important for the development of more specific perioperative protocols and patient information [12]. Standardization of postoperative pain management and focused surveillance in adolescents may decrease the risk of unanticipated read mission as proposed in recent health- services research [13]. Future prospective multicenter studies with standardized hemostatic scoring and antifibrinolytic protocols are suggested. New evidence also favours incorporation of age, indication for surgery and medication use into risk-prediction models for preoperative patient stratification [14, 15- 16]. The results of the present study add institution-related observations to these international trends.

## Conclusion:

Hemorrhage after tonsillectomy affected approximately one in twenty of these patients over the two-year period. Puberty, recurrent tonsillitis and NSAID were identified as risk factors for postoperative hemorrhage. Such results would favor use of targeted prophylaxis and more aggressive postoperative follow-up in these high-risk patients.

## Advancement to knowledge:

This study contributes contemporary (2020-2025-aligned) institutional evidence quantifying post-tonsillectomy hemorrhage incidence and confirming age ≥15 years, recurrent tonsillitis and perioperative NSAID use as independent predictors in a tertiary-care cohort, thereby reinforcing recent population-based risk models while providing locally applicable data for perioperative stratification and protocol optimization.
